# Extracellular loop 2 of G protein–coupled olfactory receptors is critical for odorant recognition

**DOI:** 10.1016/j.jbc.2022.102331

**Published:** 2022-08-01

**Authors:** Yiqun Yu, Zhenjie Ma, Jody Pacalon, Lun Xu, Weihao Li, Christine Belloir, Jeremie Topin, Loïc Briand, Jérôme Golebiowski, Xiaojing Cong

**Affiliations:** 1 Department of Otolaryngology, Eye, Ear, Nose & Throat Hospital, Ear, Nose & Throat Institute, Fudan University, Shanghai, People's Republic of China; 2 Clinical and Research Center for Olfactory Disorders, Eye, Ear, Nose & Throat Hospital, Fudan University, Shanghai, People's Republic of China; 3 School of Life Sciences, Shanghai University, Shanghai, People's Republic of China; 4 Université Côte d’Azur, CNRS, Institut de Chimie de Nice UMR7272, Nice, France; 5 Centre des Sciences du Goût et de l’Alimentation (CSGA), Université de Bourgogne-Franche Comté, CNRS, INRA, Dijon, France; 6 Department of Brain and Cognitive Sciences, Daegu Gyeongbuk Institute of Science and Technology, Daegu, South Korea; 7 Institut de Génomique Fonctionnelle, Université de Montpellier, CNRS, INSERM, Montpellier cedex 5, France

**Keywords:** olfactory receptor, molecular modeling, virtual screening, site-directed mutagenesis, ch-5HT_2C_R^ECL2^, chimeric mOR256-3 with ECL2 from the 5HT serotonin 2C receptor, ch-β_2_AR^ECL2^, chimeric mOR256-3 with ECL2 from the β2 adrenergic receptor, ch-M_2_R^ECL2^, chimeric mOR256-3 with ECL2 from the M2 muscarinic receptor, ECL, extracellular loop, FBS, fetal bovine serum, GPCR, G protein–coupled receptor, ICL, intracellular loop, MD, molecular dynamics, mOR, mouse olfactory receptor, OR, olfactory receptor, PDB, Protein Data Bank, PE, phycoerythrin, REST2, replica exchange with solute scaling 2, RTP, receptor-transporting protein, SV40, simian virus 40, TM, transmembrane helix, wt, wild-type

## Abstract

G protein–coupled olfactory receptors (ORs) enable us to detect innumerous odorants. They are also ectopically expressed in nonolfactory tissues and emerging as attractive drug targets. ORs can be promiscuous or highly specific, which is part of a larger mechanism for odor discrimination. Here, we demonstrate that the OR extracellular loop 2 (ECL2) plays critical roles in OR promiscuity and specificity. Using site-directed mutagenesis and molecular modeling, we constructed 3D OR models in which ECL2 forms a lid over the orthosteric pocket. We demonstrate using molecular dynamics simulations that ECL2 controls the shape and volume of the odorant-binding pocket, maintains the pocket hydrophobicity, and acts as a gatekeeper of odorant binding. Therefore, we propose the interplay between the specific orthosteric pocket and the variable, less specific ECL2 controls OR specificity and promiscuity. Furthermore, the 3D models created here enabled virtual screening of new OR agonists and antagonists, which exhibited a 70% hit rate in cell assays. Our approach can potentially be generalized to structure-based ligand screening for other G protein–coupled receptors that lack high-resolution 3D structures.

G protein–coupled receptors (GPCRs) are the largest family of membrane proteins in the human genome, comprising over 800 members. Half of the human GPCR genes code for olfactory receptors (ORs) (1), which can discriminate an astonishing number of different odors (2). ORs are also ectopically expressed in nonolfactory tissues, emerging as appealing drug targets (3, 4, 5, 6, 7, 8). GPCRs detect diverse ligands and control most of the cell signaling. Despite their diverse functions, GPCRs conserve a seven transmembrane helical (TM) architecture (TM1—TM7), connected by three extracellular loops (ECL1—ECL3) and three intracellular loops (ICL1—ICL3). ORs belong to class A GPCRs, which account for ∼85% of the human GPCR genes. The orthosteric ligand-binding pocket in class A GPCRs is located within the extracellular half of the TM bundle, extending ∼15 Å deep into the cell membrane (9). The pocket may be solvent accessible (*e.g.*, in receptors for peptides or soluble molecules) or shielded by ECL2 (*e.g.*, in lipid receptors and rhodopsin) (10). ECL2 is often the longest extracellular loop, which is highly variable in length, sequence, and structure (11, 12). A disulfide bond between ECL2 and TM3 is conserved in 92% of human GPCRs (13). It is important for ligand binding and receptor activation (10). Peptide-activated GPCRs mostly contain an ECL2 in the form of a β-hairpin lying on the rim of the orthosteric pocket. ECL2 of GPCRs that are modulated by small-molecule endogenous ligands exhibits diverse shapes. They are often unstructured and cover partially or fully the pocket entrance (10). Rhodopsin is a case in-between, in which a β-hairpin–shaped ECL2 inserts deep into the orthosteric pocket (14). It has been suggested that rhodopsin ECL2 represents an evolutionary transition between peptide receptors and small-molecule receptors (12). In small-molecule receptors, ECL2 may have evolved to mimic the peptide ligands and occupy part of the pocket, which renders the pocket suitable for binding small molecules. ECL2 plays important roles in ligand binding and activation of class A GPCRs (11). It may act as a gateway to the orthosteric pocket (15, 16, 17, 18, 19), bind allosteric modulators (20, 21), or participate in receptor activation (22, 23).

ECL2 of ORs are among the longest in class A GPCRs. ORs can be promiscuous or highly specific, in which ECL2 may play a central role. However, the lack of high-resolution OR structures hampers the study of OR-odorant recognition. Homology modeling combined with site-directed mutagenesis have shed light on the structure and ligand specificity of the orthosteric pocket of various ORs (24, 25, 26, 27, 28). Yet, the role and structure of ECL2 remain mostly elusive. In this work, we studied the role of ECL2 in two prototypical mouse ORs (mORs) of the same subfamily, mOR256-3 and mOR256-8, which share 54% sequence identity. Our previous work indicated that mOR256-3 is promiscuous for a series of commonly encountered odorants, whereas mOR256-8 is rather specific (29). In this study, we found that ECL2 properties strongly modulate OR-odorant recognition. We performed site-directed mutagenesis along ECL2 and built 3D OR models that are in concordance with the mutagenesis data. Virtual screening using the 3D models identified new mOR256-3 ligands, including an antagonist that inhibited some of the agonists. The 3D models provide structural explanations to the promiscuity of mOR256-3 and the selective antagonism.

## Results

### Sequence analysis of OR ECL2

Sequence alignment of 1521 human and mORs showed that their ECL2 mostly contain 34 to 35 amino acids (Fig. S1). They are longer than ECL2 in most class A GPCRs. Three cysteines are highly conserved (C169, C179, and C189 in mOR256-3, conserved in 93.4%, 99.5%, and 95.0% of human and mORs, respectively). C179 forms the classic disulfide bond with TM3, whereas C169 and C189 have been suggested to form a second disulfide bond within ECL2 (30). A few residues around the two disulfide bonds are highly conserved, whereas the rest of the OR ECL2 sequence displays low conservation (Fig. S1). It is plausible that the two disulfide bonds are important for ECL2 structuring and OR functions.

### Nonspecific roles of ECL2 in OR responses to odorants

In our previous work, we screened diverse odorants at a near-saturating concentration (300 μM) on several ORs in the heterologous Hana3A cells. We found a wide range of potential ligands for mOR256-3 but only two for mOR256-8 (29). Yet, one or few point mutations in mOR256-8 could significantly expand its ligand spectrum (29). Here, we reexamined 20 of these odorants at various concentrations in Hana3A cells expressing mOR256-3 or mOR256-8. Ten odorants activated mOR256-3 in a dose-dependent manner, including cyclic and acyclic alcohols, aldehydes, acids, ketones, and esters: R-carvone, coumarin, 1-octanol, allyl phenylacetate, benzyl acetate, citral, geraniol, 2-heptanone, octanal, and octanoic acid (Table S1 and Fig. S2*A*). mOR256-8 responded only to 1-octanol and geraniol in a dose-response manner, which are two primary acyclic alcohols of similar lengths (Table S1 and Fig. S2*B*).

**Figure 1:**
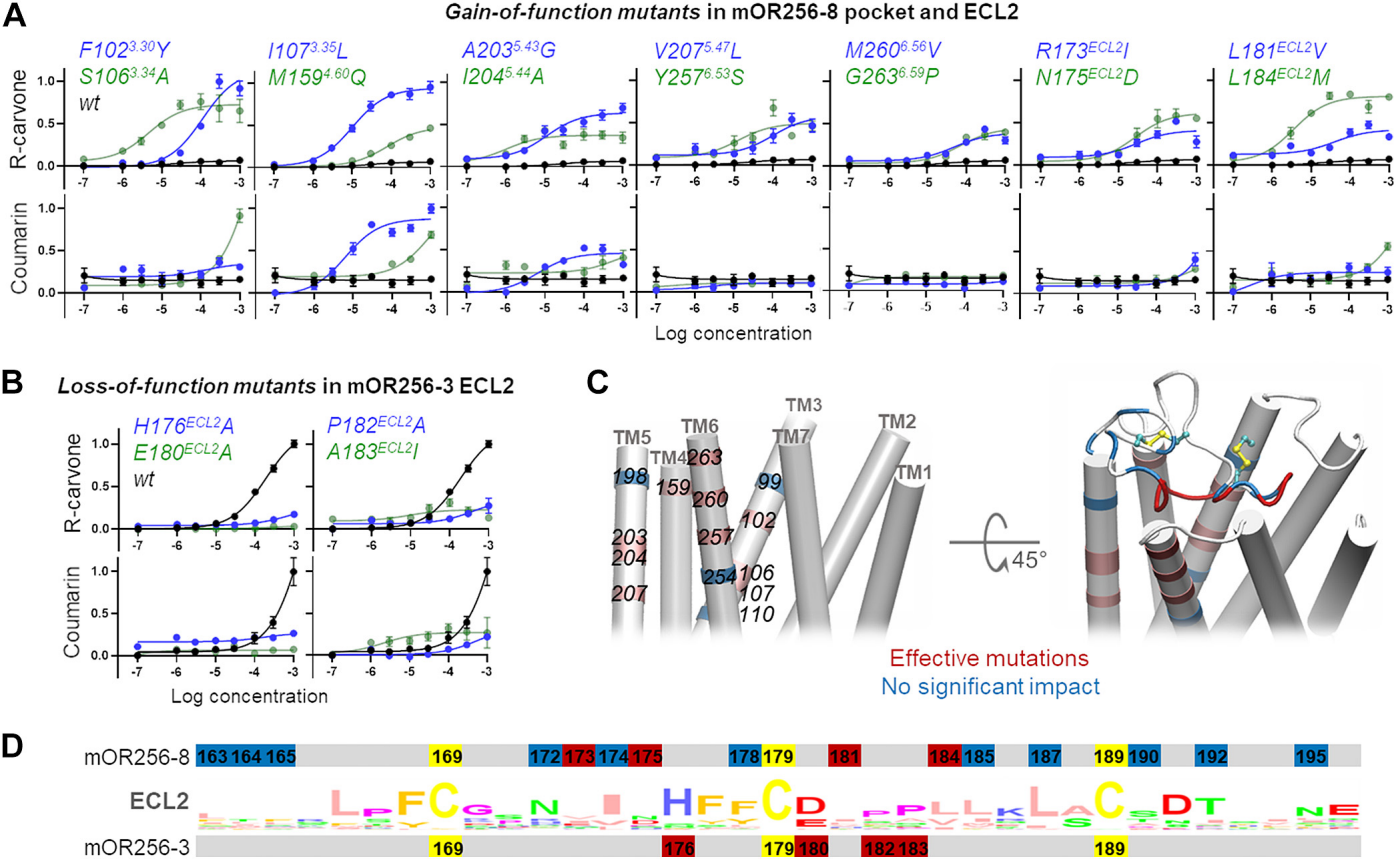
**Site-directed mutagenesis and location of the mutation sites.** Mutations in (*A*) mOR256-8 pocket and ECL2 and (*B*) mOR256-3 ECL2 affected the response to various odorants. Data are mean ± SEM of three independent experiments. *C*, homology model of mOR256-3 selected according to the data in *A* and *B*. *D*, consensus ECL2 sequence of human and mouse ORs and location of the mutation sites. Effective mutations are colored in *pink* (in the pocket) or *red* (in ECL2). Noneffective mutations are colored in *blue*, including V99^3.27^A, V110^3.38^T, L198^5.38^E, S254^6.50^T, R172^ECL2^N, I174^ECL2^L, L178^ECL2^F, I185^ECL2^L, M187^ECL2^L, V190^ECL2^T, A192^ECL2^T, and V195^ECL2^N in mOR256-8. In the 3D models, consistently, the noneffective mutation sites (*blue*) do not constitute the ligand-binding site or the pathway to the binding site. ECL2, extracellular loop 2.

Focusing on the role of ECL2, we performed site-directed mutagenesis to probe the residues that are responsible for the functional differences between mOR256-3 and mOR256-8. Based on the 3D models in our previous work (29, 31, 32, 33, 34), we mutated 14 residues on TM3–TM6 around the orthosteric pocket, as well as 15 residues in ECL2 of mOR256-8 that differ from mOR256-3. In the narrowly tuned mOR256-8, these residues were mutated one by one into their counterpart in the broadly tuned mOR256-3. We then tested the response of the mutant receptors to R-carvone and coumarin, two reference ligands of mOR256-3. While wild-type (wt) mOR256-8 does not respond to these odorants, 14 of the mutants showed dose-dependent responses to R-carvone, and some of them also responded to coumarin (Fig. 1*A*). Four of the mutations were in ECL2, R173I, N175D, L181V, and L184M (Fig. 1*A*). These residues flank the ECL2–TM3 disulfide bond, suggesting that this region (residues 173–184) is important for the receptor function. Five residues in this region are conserved in mOR256-8 and mOR256-3 (H176, F177, E180, P182, and A183). Therefore, we mutated these five residues in mOR256-3 to evaluate their role in this promiscuous receptor. They were mutated into alanine, except for A183, which was mutated into a bulky isoleucine. While F177A impaired receptor expression on the cell surface (Fig. S3), the other four mutations systematically diminished the receptor’s response to R-carvone and coumarin (Fig. 1*B*). The aforementioned mutations in the two receptors had less drastic impacts on the response to geraniol (Fig. S4), which suggest that geraniol interacts with the receptors in a different manner.

We also generated a chimeric mOR256-8 in which ECL2 was replaced with that of mOR256-3. However, it did not gain response to the ligands of mOR256-3. The aforementioned data highlight that residues 173–184 in ECL2 are critical but not solely responsible for ligand recognition or receptor promiscuity. This is in line with the notion that in class A GPCRs, ECL2 acts as a vestibule or a molecular sieve of ligand binding and/or an allosteric site of receptor activation. Since residues 173–184 in ORs surround the conserved ECL2–TM3 disulfide bond, they are likely important in most, if not all, mammalian ORs. For instance, mutations in this region have dramatic impact on the response of mOR-EG to its odorants (28). This region has also been found to interact with the orthosteric ligands in several nonolfactory class A GPCRs (11).

### 3D modeling explains OR promiscuity

**Figure 2:**
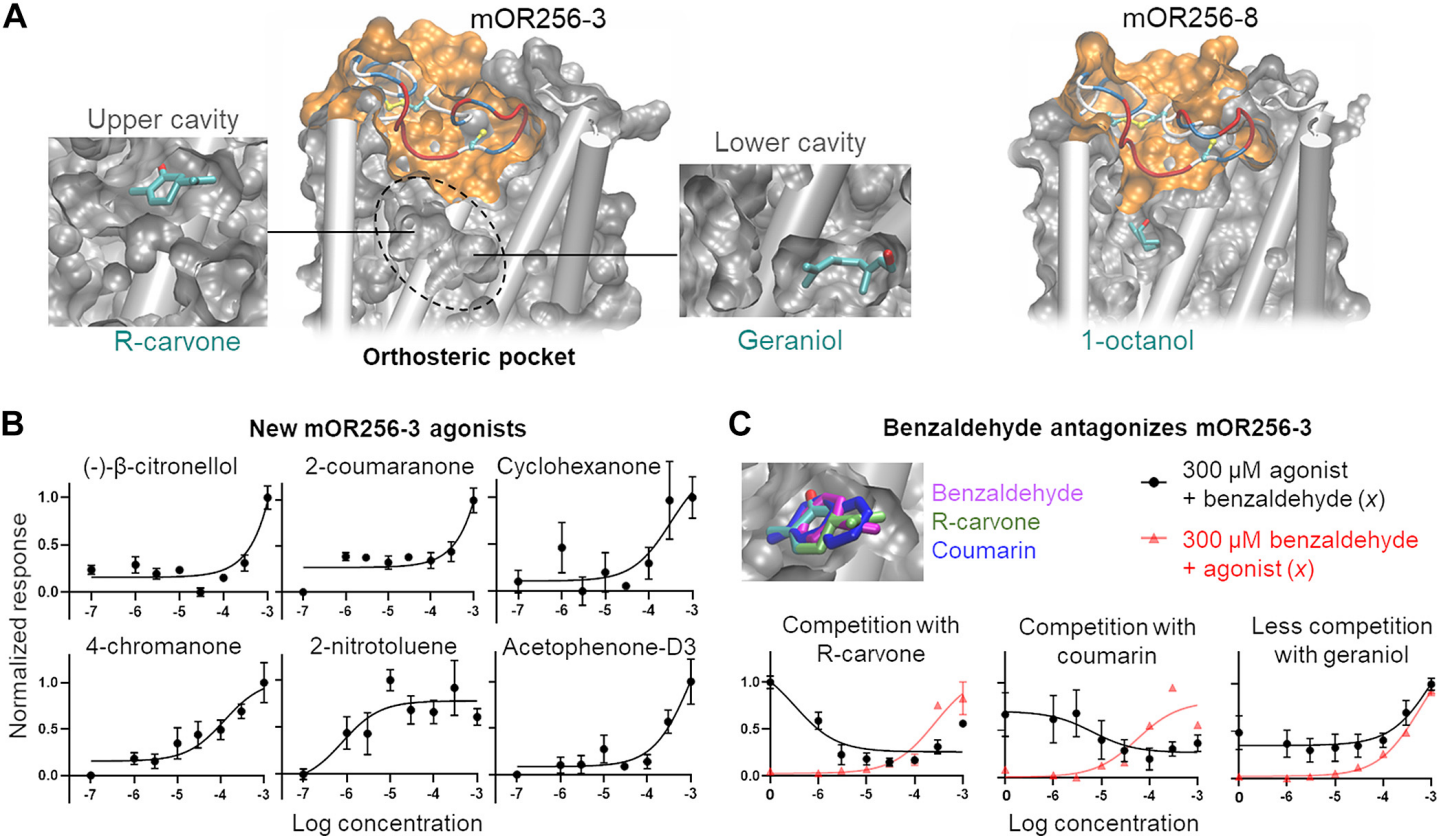
**Selected 3D models and new mOR256-3 ligands discovered by virtual screening.***A*, cross-section of the best model of mOR256-3 and mOR256-8, illustrating ECL2 as the pocket lid. mOR256-3 displays two connected cavities in the pocket, in which the *upper cavity* binds cyclic ligands and the *lower one* accommodates acyclic molecules. *B*, dose-dependent curves of new mOR256-3 agonists from virtual screening. *C*, benzaldehyde binds in the same cavity as R-carvone and coumarin. It inhibits R-carvone, coumarin, and geraniol. Data are mean ± SEM of three independent experiments. ECL2, extracellular loop 2.

To date, there are no high-resolution OR structures or structural information on the structural fold of OR ECL2. We generated three types of 3D models using AlphaFold 2 (DeepMind Technologies) (35), Modeller (University of California San Francisco) (36), and SWISS-MODEL (Swiss Institute of Bioinformatics) (37). The three models displayed distinct structures in ECL2 (Figs. 1*C* and S5). We evaluated the predictivity of the models using site-directed mutagenesis data and docking. The model that best matched these data was generated by Modeller based on our hand-curated multiple sequence alignment (Fig. S6). In this model, ECL2 appears as an unstructured coil, in which residues 173–184 form a lid of the orthosteric pocket (Fig. 1*C*). Residues 180–183 may interact directly with the ligands (Fig. 1*C*). The model also suggests that the pocket of mOR256-3 is much larger than mOR256-8, showing two connected cavities (Fig. 2*A*). This may allow mOR256-3 to accommodate odorants of diverse size and shape. Molecular docking suggests that the upper cavity can accommodate the cyclic ligands, whereas the deeper cavity accommodates the acyclic ones (Figs. 2*A* and S7). The pocket of mOR256-8 shows only one small cavity for its acyclic ligands. We estimated the pocket volume of all the human and mORs by summing up the side-chain volume of the residues outlining the pocket with or without ECL2. We found that the pocket size of mOR256-3 is ranked in the 47th and 46th percentile with and without ECL2, respectively, whereas that of mOR256-8 is at the 26th and 22nd, respectively (Fig. S8). Thus, the larger pocket volume of mOR256-3 than mOR256-8 may provide a structural explanation to the promiscuity of the former. In order to assess this hypothesis and the model predictivity, we use the model to virtually screen for new mOR256-3 ligands by molecular docking.

Docking benchmarks were first performed with 52 compounds, including 10 known ligands of mOR256-3 and 42 decoys (Table S2) (29). An ensemble-docking protocol (Fig. S9) was used to account for the conformational flexibility of the OR. Namely, enhanced sampling molecular dynamics (MD) simulations were performed on the initial model of mOR256-3 to sample the receptor conformations (see the Experimental procedures section for details). Ten receptor conformers (snapshots) were extracted from a clustering analysis of the MD trajectory. The 52 benchmark compounds were docked to each of the 20 conformers using AutoDock Vina (The Scripps Research Institute) (38) and ranked by their Vina scores for the given conformer. The “best” conformers were chosen as those that could best separate the ligands from the decoys by the Vina scores (Fig. S9). We performed this benchmarking process for our in-house model as well as for the models generated by AlphaFold 2 and SWISS-MODEL. The in-house model—generated by Modeller and selected according to site-directed mutagenesis data—gave the best predictions on the benchmark compounds (Table 1). Removing ECL2 from this model significantly reduced the predictivity (Table 1).

**Table 1:** Docking benchmark using different 3D models of mOR256-3 and 52 compounds

Model	MD snapshot^a^	MCC	Hit rate^b^(precision)	Recall^c^	True positive	True negative	False positive	False negative
In-house model	1	0.50	0.60	0.60	6	38	4	4
	2	0.26	0.40	0.40	4	36	6	6
In-house model without ECL2	1	0.26	0.40	0.40	4	36	6	6
	2	0.13	0.30	0.30	3	35	7	7
SWISS-MODEL	1	0.23	0.36	0.40	4	35	7	6
	2	0.20	0.33	0.40	4	34	8	6
AlphaFold 2	1	0.38	0.50	0.50	5	37	5	5
	2	0.26	0.40	0.40	4	36	6	6

a Two snapshots that gave the best Matthew’s correlation coefficient (MCC) as a statistical measure of the model’s predictivity (64). MCC returns a value between −1 (total disagreement between prediction and observation) and +1 (perfect prediction).

b Hit rate or precision, the fraction of true ligands among the model predicted ones.

c Recall indicates the fraction of true ligands retrieved by the model out of all the true ligands in the benchmark compounds.

Finally, we chose two best conformers of the aforementioned in-house model to virtually screen a library of 80 odorants in our laboratory (Tables S3 and S4). The screening returned 10 candidate compounds (Table S3), which were tested in functional assays in Hana3A cells. Six of them turned out to be mOR256-3 agonists and one (benzaldehyde) was an antagonist, giving 70% hit rate (Fig. 2*B* and *C* and Table S3). Benzaldehyde antagonized R-carvone, coumarin, and geraniol (Fig. 2*C*). Docking predicted that benzaldehyde may bind in the upper cavity of the mOR256-3 pocket for cyclic ligands, similar to R-carvone and coumarin (Fig. 2*C*).

**Figure 3:**
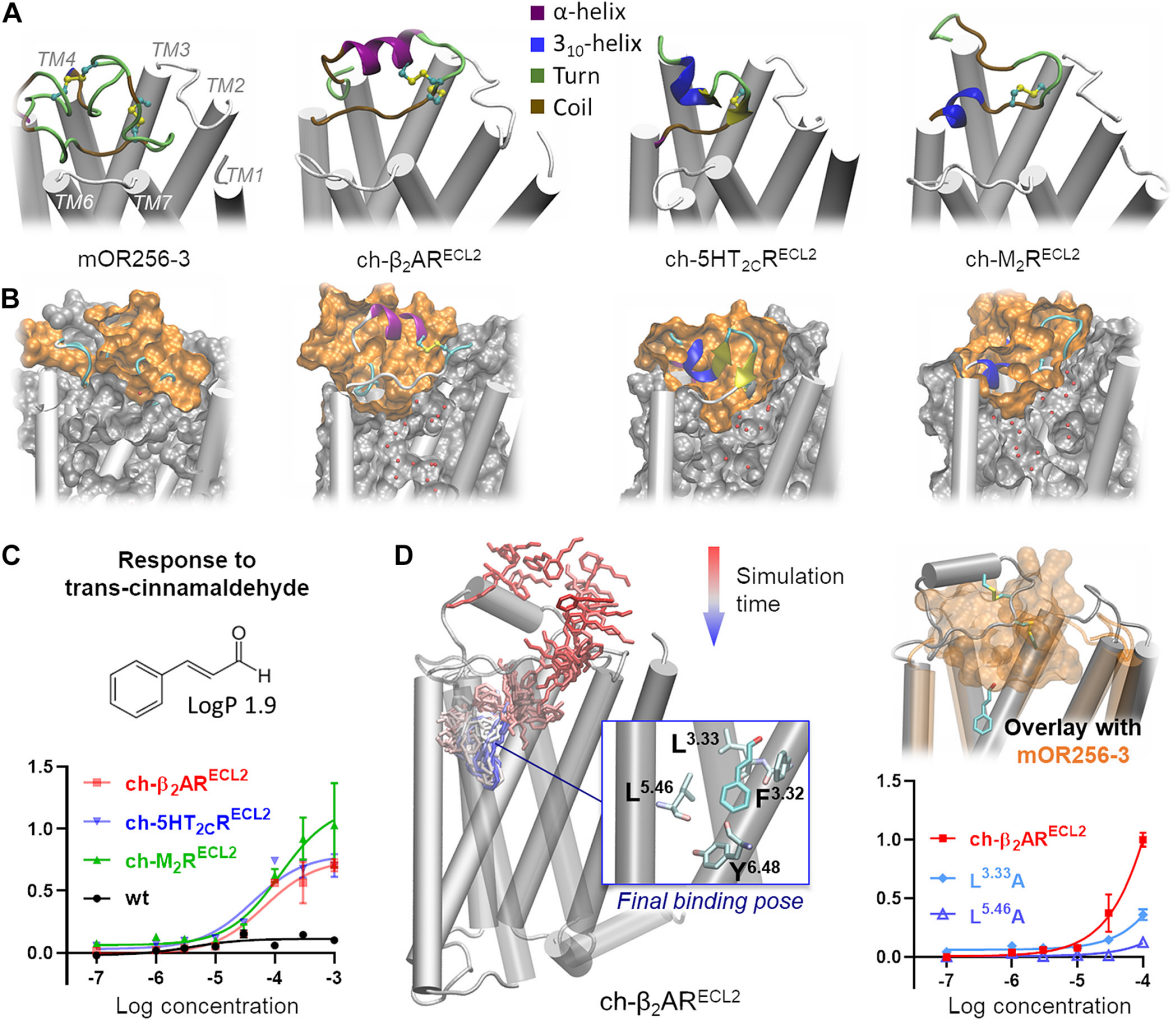
**Structure models and functional assays of mOR256-3 chimeras.***A*, homology models of mOR256-3 variants with different ECL2 sequences and structures (in *cartoon* presentation, colored by secondary structure). *B*, the pocket of the chimeras was hydrated during molecular dynamics (MD) simulations without ligand in the pocket, whereas that of wt mOR256-3 remained dehydrated during the same simulation course. Shown here is the final MD simulation frame in cross-section. The water molecules within the pocket are shown in *red balls*, and the surface of ECL2 is shown in *orange*. *C*, dose-dependent responses of the three chimeras to transcinnamaldehyde. *D*, transcinnamaldehyde entered the pocket of β_2_AR^ECL2^*via* the ECL2–TM7 gap during MD simulations. It adopted a binding pose that interacts with the toggle switch Y^6.48^. Mutating the transcinnamaldehyde-binding residues L^3.33^ and L^5.46^ diminished the receptor response to this ligand. An overlay with wt mOR256-3 (*orange*) shows a steric clash of transcinnamaldehyde with ECL2, which is likely the reason why wt mOR256-3 does not respond to this odorant. Data are mean ± SEM of three independent experiments. ECL2, extracellular loop 2; TM, transmembrane.

### ECL2 controls pocket shape and hydrophobicity

To further examine the role of ECL2 in odorant recognition, we constructed three mOR256-3 chimeras, by replacing its ECL2 with that of M2 muscarinic receptor, β2 adrenergic receptor, and 5HT serotonin 2C receptor, respectively (denoted as ch-β_2_AR^ECL2^, ch-M_2_R^ECL2^, and ch-5HT_2C_R^ECL2^). ECL2 of these receptors exhibit distinct structures (Fig. 3*A*). In Hana3A cells, the chimeras showed no significant response to the mOR256-3 ligands (Fig. S10). Nevertheless, they all displayed specific dose-dependent response to transcinnamaldehyde (Fig. 3*B*), whereas wt mOR256-3 does not respond to this odorant (29). To understand how the chimeric mOR256-3 became specific receptors of transcinnamaldehyde, we built homology models for the chimeras and performed all-atom MD simulations in an explicit membrane–water environment. The homology models were built by assuming that ECL2 of the chimeras preserve the same fold as in β_2_AR, M_2_R, and 5HT_2C_R, respectively. The models illustrated that ECL2 of the chimeras only partly covered the ligand entrance. The orthosteric pocket of the chimeras was hydrated during the MD, whereas that of wt mOR256-3 was shielded from hydration by ECL2 (Fig. 3*A*). This might be the reason why the chimeras did not respond to the hydrophobic ligands of mOR256-3. Rather, they responded to the less hydrophobic transcinnamaldehyde (Fig. 3*C*).

We then added transcinnamaldehyde in the MD simulations of wt mOR256-3 and the chimeras to monitor the ligand binding. The ligand was initially placed at 10 Å above ECL2 and was restrained within a 15 Å radius around ECL2. Each system underwent 30 independent MD runs of 200 ns. We observed two binding events in ch-β_2_AR^ECL2^, in which transcinnamaldehyde entered the orthosteric pocket near the toggle switch residue Y6.48 (Fig. 3*D*). It caused the side chain of Y6.48 to flip toward TM5, which is likely an early step of OR activation (32). In the case of wt mOR256-3, ch-M_2_R^ECL2^, and ch-5HT_2C_R^ECL2^, transcinnamaldehyde associated with ECL2 but could not enter the pocket. The binding pose of transcinnamaldehyde in ch-β_2_AR^ECL2^ suggests that wt mOR256-3 cannot accommodate this ligand, since ECL2 occupies part of its pocket (Fig. 3*D*). Indeed, mOR256-3 ligands are generally smaller or more flexible than transcinnamaldehyde. The lack of ligand binding in ch-M_2_R^ECL2^ and ch-5HT_2C_R^ECL2^ was likely because of insufficient sampling of the ECL2 conformations in these very short simulations. The entrance to the pocket is narrower in the initial models in ch-M_2_R^ECL2^ and ch-5HT_2C_R^ECL2^ than that in ch-β_2_AR^ECL2^. To verify the binding pose of transcinnamaldehyde observed in the MD simulations, we mutated three pocket residues that are in close contact with the ligand. Mutations L^3.33^A and L^5.46^A abolished the receptor response to transcinnamaldehyde (Fig. 3*D*). F^3.32^A impaired the receptor expression on the cell surface (Fig. S3) and is thus not discussed. The results suggest that the recognition of transcinnamaldehyde is specific to the orthosteric pocket, whereas ECL2 served as an unspecific molecular sieve for the ligand entrance.

## Discussion

Mammalian OR sequences have highly diversified during evolution to detect and discriminate a vast spectrum of odorants. Specific (or narrowly turned) ORs may be responsible for the detection of specific odorants or endogenous ligands when ectopically expressed in nonolfactory tissues (3, 4, 5, 6). Promiscuous (or broadly tuned) ORs may play exert important functions in olfaction, such as expanding the detection spectrum, diversifying the combinatorial code, and acting as general odor detectors or odor intensity analyzers (29). Promiscuous ORs feature mostly nonpolar interactions in the orthosteric pocket with odorants, which are more adaptable to different odorant structures (33, 39). Here, we showed that ECL2 is indispensable for OR promiscuity. ECL2 acts as a pocket lid to maintain the pocket hydrophobicity and also forms the upper part of the pocket to control its shape and volume. Its structural flexibility and mostly hydrophobic nature may tolerate diverse odorants, resulting in promiscuity. Indeed, in class A GPCRs, ECL2 may change conformations upon ligand binding and adopt different forms for different ligands (11). The evolution of ECL2 in class A GPCRs is strongly coupled to that of the orthosteric pocket (12). Therefore, class A GPCR–ligand recognition relies on the interplay between ECL2 and the orthosteric pocket. ECL2 may also take part in receptor activation *via* allosteric coupling with the receptor movements on the intracellular side (11). However, this aspect is beyond the scope of the current study. Note that the 3D models reported here are not to present the exact structural fold of ECL2. Rather, they are to illustrate the approximate position of the ECL2 residues according to the mutagenesis data. Since mOR256-3 ECL2 features mostly nonpolar interactions with the odorants, such approximate models serve as suitable structural basis for ligand discovery, as demonstrated by the virtual screening performance. The MD simulations based on these models are insufficient to sample the ECL2 conformational changes upon ligand binding. High-resolution OR structures may enable further investigations on this challenging question. Nevertheless, the models provide an explanation to competitive antagonism, which has been shown to be essential for the perception of odor mixtures (40). Therefore, the models and the virtual screening approach established here may serve the design of biosensors with wide odor detection spectrum or specific odor maskers and/or drug candidates targeting ectopic ORs in nonolfactory tissues.

## Experimental procedures

### Chemicals and OR constructs

Odorants were purchased from Sigma–Aldrich. They were dissolved in dimethyl sulfoxide to make stock solutions at 1 mM and then diluted freshly in optimal MEM (Thermo Fisher Scientific) to prepare the odorant stimuli. The OR constructs were kindly provided by Dr Hiroaki Matsunami (Duke University). Site-directed mutants were constructed using the Quikchange site-directed mutagenesis kit (Agilent Technologies). The sequences of all plasmid constructs were verified by both forward and reverse sequencing (Sangon Biotech).

### Chimera construction

All chimeras were constructed by three PCR steps with modification (41). Briefly, two fragments were amplified from the mOR256-3, whereas ECL2 of β_2_AR, M_2_R, and 5HT_2C_R was synthesized by Sangon Biotech Co. The primers were partially complementary at their 5′ ends to the adjacent fragments, necessary to fuse the different fragments together. Three fragments were purified and fused together in a second PCR step. Equal amount of each fragment was mixed with dNTP and Phusion High-Fidelity DNA Polymerase (NEB) in the absence of primers. The PCR program consisted of 10 repetitive cycles with a denaturation step at 98 °C for 10 s, an annealing step at 55 °C for 30 s, and an elongation step for 30 s at 72 °C. The third step corresponded to the PCR amplification of the fusion product using the primers of mOR256-3. The PCR product was purified and ligated into PCI vector. The sequences of all chimeras were verified by both forward and reverse sequencing.

### Cell culture and transfection

We used Hana3A cells, a human embryonic kidney 293T–derived cell line that stably expresses receptor-transporting proteins (RTP1L and RTP2), receptor expression–enhancing protein 1 (REEP1), and olfactory G protein (Gα_olf_) (42). The cells were grown in MEM (Corning) supplemented with 10% (v/v) fetal bovine serum (FBS; Thermo Fisher Scientific) plus 100 μg/ml penicillin–streptomycin (Thermo Fisher Scientific), 1.25 μg/ml amphotericin (Sigma–Aldrich), and 1 μg/ml puromycin (Sigma–Aldrich).

All constructs were transfected into the cells using Lipofectamine 2000 (Thermo Fisher Scientific). Before the transfection, the cells were plated on 96-well plates (NEST) and incubated overnight in MEM with 10% FBS at 37 °C and 5% CO_2_. For each 96-well plate, 2.4 μg of pRL-SV40 (simian virus 40), 2.4 μg of CRE-Luc, 2.4 μg of mouse RTP1S, and 12 μg of receptor plasmid DNA were transfected. The cells were subjected to a luciferase assay 24 h after transfection.

### Luciferase assay

The luciferase assay was performed with the Dual-Glo Luciferase Assay Kit (Promega) following the protocol (42). OR activation triggers the Gα_olf_-driven AC-cAMP-PKA signaling cascade and phosphorylates cAMP response element–binding protein. Activated cAMP response element–binding protein induces luciferase gene expression, which can be quantified luminometrically (measured here with a bioluminescence plate reader [MD SPECTRAMAX L]). Cells were cotransfected with firefly and *Renilla* luciferases where firefly luciferase served as the cAMP reporter. *Renilla* luciferase is driven by a constitutively active SV40 promoter (pRL-SV40; Promega), which served as a control for cell viability and transfection efficiency. The ratio between firefly luciferase *versus Renilla* luciferase was measured. Normalized OR activity was calculated as (*L*_N_ − *L*_min_)/(*L*_max_ − *L*_min_), where *L*_N_ is the luminescence in response to the odorant, and *L*_min_ and *L*_max_ are the minimum and maximum luminescence values on a plate, respectively. The assay was carried out as follows: 24 h after transfection, medium was replaced with 100 μl of odorant solution (at different doses) diluted in Optimal MEM, and cells were further incubated for 4 h at 37 °C and 5% CO_2_. After incubation in lysis buffer for 15 min, 20 μl of Dual-Glo Luciferase Reagent was added to each well of 96-well plate, and firefly luciferase luminescence was measured. Next, 20 μl Stop-Glo Luciferase Reagent was added to each well, and *Renilla* luciferase luminescence was measured. Data analysis followed the published procedure (42). Three-parameter dose–response curves were fitted with GraphPad Prism 9 (GraphPad Software, Inc).

### Flow cytometry analysis

Hana3A cells were seeded in 35 mm dishes. The cells were cultured overnight to >80% confluence and transfected with 0.3 μg RTP1S, 0.3 μg GFP, and 0.8 μg OR plasmid by Lipofectamine 2000. At 24 h after transfection, the cells were stripped with TrypLE Express Enzyme (Thermo Fisher Scientific) and then kept in round bottom polystyrene tubes on ice. The cells were spun down at 200*g* for 3 min at 4 °C and resuspended in PBS containing 2% FBS and 15 mM NaN_3_. They were incubated with primary antibody mouse antirhodopsin for 45 min and then with phycoerythrin (PE)-conjugated donkey antimouse immunoglobulin G (Jackson ImmunoResearch; catalog no.: 715-116-150) in the dark for 30 min on ice. After washing twice, the cells were analyzed using Beckman Coulter CytoFLEX with gating for GFP positive, single, viable cells. The measured PE fluorescence intensities were analyzed and visualized using FlowJo (BD), version 10. The PE fluorescence intensity was normalized to the average value of wt ORs for statistical analysis.

### Molecular modeling

The in-house models of mOR256-3 and mOR256-8 were generated with Modeller 9.21 (36) using our hand-curated sequence alignment to four structure templates: human a2AR (Protein Data Bank [PDB] ID: 2YDV), human CXCR1 (PDB ID: 2LNL), human CXCR4 (PDB ID: 3ODU), and bovine rhodopsin (PDB ID: 1U19). The N and C termini were excluded. The template structures are all in inactive state. The sequence similarity between the templates and the two target ORs ranged from 31% to 38%. In the TM regions, the sequence similarity was 38–44%. For the three chimeras, the ECL2 structure of β_2_AR (PDB ID: 2RH1), M_2_R (PDB ID: 3UON), and 5HT_2C_R (PDB ID: 6BQH), respectively, was used as templates for the ECL2. For each receptor, 2500 models were generated and ranked by the DOPE score (43). The 250 top ranked models were selected and clustered using the k-means algorithm. We obtained five clusters for each receptor and selected a representative model that was the most compatible with the mutagenesis data. The SWISS-MODELS were generated using the SWISS-MODEL webserver (37) and the target OR sequence. Template search and model building were performed using default settings of the webserver. The AlphaFold 2 models (35) were generated using the API hosted at the Söding Laboratory based on the MMseqs2 server (44). Using the target OR sequence as input, the models were generated using the parameters (35). Docking was performed with AutoDock Vina (38). The receptors were prepared with AutodockTools to add nonpolar hydrogens and Gasteiger charges. A grid box was set to encompass the pocket and the lid, with a 0.375 Å grid point spacing. Initial 3D coordinates of the ligands were generated using Balloon (Åbo Akademi University) (45) and converted by AutoDock Raccoon (The Scripps Research Institute) for the docking (46). Pocket residues and ligand rotatable bonds were set flexible. For virtual screening, however, pocket residues were kept rigid and multiple receptor conformers were used. Other parameters for the docking were left as their default values.

### MD simulations

The receptor N and C termini were truncated at residues 23 and 305, respectively. Protonation state of titratable residues in the receptors were predicted at pH 7 using the H++ server (47). The receptors or receptor-odorant complexes were embedded in a bilayer of 1-palmitoyl-2-oleoyl-*sn*-glycero-3-phosphocholine using PACKMOL-Memgen (Heinrich Heine University Düsseldorf) (48). Each system was solvated in a periodic 75 × 75 × 105 Å^3^ box of explicit water and neutralized with 0.15 M of Na^+^ and Cl^−^ ions. Effective point charges of the ligands were obtained by restrained electrostatic potential fitting (49) of the electrostatic potentials calculated with the HF/6-31G∗ basis set using Gaussian 09 (50). The Amber 14SB (51), lipid 14 (52), and GAFF (53) force fields were used for the proteins, lipids, and ligands, respectively. The TIP3P model (54) and the Joung–Cheatham parameters (55) were used for the water and the ions, respectively.

The process of ligand binding was simulated with 30 runs of 200 ns of all-atom brute-force MD for each OR–ligand pair using Amber18. The ligand was initially placed 10 Å above ECL2. After energy minimization, each system was gradually heated to 310 K with a restraint of 200 kcal/mol on the receptor and ligand. This was followed by 5 ns of pre-equilibration with a restraint of 5 kcal/mol and 5 ns of unrestrained equilibration. Bonds involving hydrogen atoms were constrained using the SHAKE algorithm (56), allowing for a 2-fs time step. van der Waals and short-range electrostatic interactions were cut off at 12 Å. Long-range electrostatic interactions were computed using particle mesh Ewald (57) method with a Fourier grid spacing of 1.2 Å. During the production run, when the ligand exceeded 15 Å from the center of ECL2, a distance restraint of 10 kcal/mol was applied to drive the ligand toward the center. Finally, the trajectories were visualized with VMD 1.9.2 (University of Illinois Urbana-Champaign) to inspect the binding events.

To thoroughly sample the conformations of mOR256-3 for ensemble docking, we used an enhanced sampling technique, replica exchange with solute scaling 2 (REST2) (58). REST2 MD was performed with 48 replicas in the *NVT* ensemble using Gromacs 5.1 (University of Groningen, Uppsala Universitet) (59) patched with the PLUMED 2.3 plugin (the PLUMED consortium) (60). The protein and ligands were considered as “solute” in the REST2 scheme. The force constants van der Waals, electrostatic, and dihedral terms of the protein and ligands were scaled down to facilitate conformational changes. The effective temperatures used for generating the REST2 scaling factors ranged from 310 to 700 K, following a distribution calculated with the Patriksson–van der Spoel approach (61). Exchange between replicas was attempted every 1000 simulation steps. This setup resulted in an average exchange probability of ∼40%. A total of 60 ns × 48 replicas of REST2 MD was carried out. The first 10 ns were discarded for equilibration, and only the original unscaled replica (at 310 K effective temperature) was collected. The Gromacs clustering tool was used to analyze the simulation trajectory. An RMSD-based clustering was performed on the Cα atoms using the GROMOS method (62) and a 1 Å cutoff. The representative frames of the top 20 clusters (covering 97% of the trajectory) were extracted for ensemble docking.

## Data availability

All data generated or analyzed during this study are included in this published article and its supporting information files.

## Supporting information

This article contains supporting information including Tables S1—S4 and Figures S1—S10. MD simulation trajectories of mOR256-3 and transcinnamaldehyde binding to ch-β_2_AR^ECL2^ are available at https://mycore.core-cloud.net/index.php/s/MkGBt36XDBbMeGz. Only the protein and ligand are shown for clarity (29, 34, 63).

## Conflict of interest

The authors declare that they have no conflicts of interest with the contents of this article.
