# Supplementary material for: Extracellular loop 2 of G protein–coupled olfactory receptors is critical for odorant recognition
Source: J Biol Chem. 2022 Aug 1;298(9):102331. doi: 10.1016/j.jbc.2022.102331 (PMC9442423; doi:10.1016/j.jbc.2022.102331)
Supplement: Supporting information [file mmc1.pdf]

Extracellular loop 2 of G protein-coupled olfactory receptors is critical for odorant recognition

Yiqun Yu[1,2,‡,], Zhenjie Ma[3‡], Jody Pacalon[4‡], Lun Xu[1], Weihao Li[1], Christine Belloir[5], Jeremie Topin[4], Loïc Briand[5], Jérôme Golebiowski[6]*, Xiaojing Cong[7]**

[1] Ear, Nose & Throat Institute, Department of Otolaryngology, Eye, Ear, Nose & Throat Hospital, Fudan University, Shanghai 200031, People's Republic of China

[2] Clinical and Research Center for Olfactory Disorders, Eye, Ear, Nose & Throat Hospital, Fudan University, Shanghai 200031, People's Republic of China

[3] School of Life Sciences, Shanghai University, Shanghai 200444, People's Republic of China

[4] Université Côte d'Azur, CNRS, Institut de Chimie de Nice UMR7272, 06108 Nice Cedex 2, France

[5] Centre des Sciences du Goût et de l'Alimentation (CSGA), Université de Bourgogne-Franche Comté, CNRS, INRA, 21000 Dijon, France

[6] Department of Brain and Cognitive Sciences, Daegu Gyeongbuk Institute of Science and Technology, Daegu 711-873, South Korea

[7] Institut de Génomique Fonctionnelle, Université de Montpellier, CNRS, INSERM, 34094 Montpellier Cedex 5, France.

‡These authors contributed equally.

* Correspondence should be addressed to yu_yiqun@fudan.edu.cn, jerome.golebiowski@gmail.com or xiaojing.cong@igf.cnrs.fr.

Table S1. Structure, hydrophobicity and potency of mOR256-3-wt ligands.

Odorant	Structure	LogP[a]	Odorant	Structure	LogP[a]
R-carvone	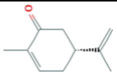	2.7	1-octanol	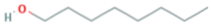	3.0
Coumarin	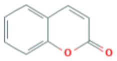	2.4	Octanal	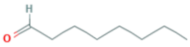	3.5
Allyl phenylacetate	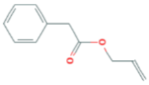	~2.4[b]	Octanoic acid	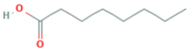	3.1
Benzyl acetate	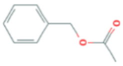	2.0	Citral	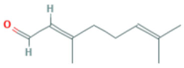	3.5
2-heptanone	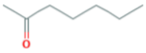	2.0	Geraniol	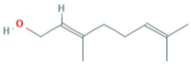	3.6

[a] data from PubChem

[b] computed by XLogP3

Table S2. Decoy[a] compounds used for docking benchmark.

Odorant	PubChem CID	Odorant	PubChem CID
R-(+)-Pulegone	442495	Diethyl sebacate	8049
1-Butaol	263	d-limonene	440917
2,3-Hexanedione	19707	Ethaol	702
2,5-Dimethylpyrazine	31252	Ethyl acetate	8857
2,5-Dimethylpyrrole	12265	Eugenol	3314
Ambrette	6753	Furfural	7362
2-Methoxy-4-methylpheol	7144	Hexyl octaoate	14228
2-Octaone	8093	Isobutylamine	6558
3-Methyl-2-butaol	11732	Isobutyraldehyde	6561
Acetaldehyde	177	Isobutyric acid	6590
Acetopheone	7410	Lilial	228987
Allyl hexaoate	31266	lyral	91604
Ammonium hydroxide	14923	m-Cresol	342
Amyl butyrate	10890	Musk ketone	6669
Amyl laurate	62571	Propyl acetate	7997
α-Phellandrene	7460	Pyridine	1049
Benzyl alcohol	244	Pyrrolidine	31268
Benzyl salicylate	8363	Thymol	6989
Phenethylamine	1001	Toluene	1140
Cyclohexylamine	7965	Cinnamaldehyde	637511
Diacetyl	650	Triethylamine	8471

[a] Non-effective compounds from the single-dose screening in ref. (29), except for 5 compounds which showed activities in dose-dependent assays in ref. (64) and this work.

Table S3. Ten candidate compounds selected from virtual screening for functional assays. The compounds were selected using the procedure shown in Fig. S9.

Name	PubChem CID	Structure	SMILES
Cyclohexanone	7967	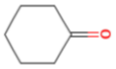	C1CCC(=O)CC1
(-)-β-Citronellol	7793	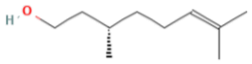	C[C@@H](CCC=C(C)C)CCO
2-Coumaranone	68382	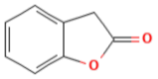	C1C2=CC=CC=C2OC1=O
Ethyl vanillin	8467	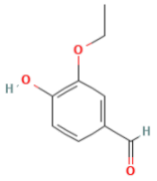	CCOC1=C(C=CC(=C1)C=O)O
2,4-DNT	8461	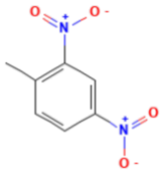	CC1=C(C=C(C=C1)[N+](=O)[O-])[N+](=O)[O-]
2-Ethyl fenchol	106997	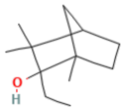	CCC1(C(C2CCC1(C2)C)(C)C)O
Acetophenone-D3	140244	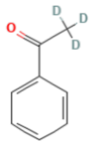	[2H]C([2H])([2H])C(=O)C1=CC=CC=C1
4-chromanone	68110	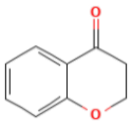	C1COC2=CC=CC=C2C1=O
2-Nitrotoluene	6944	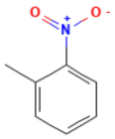	CC1=CC=CC=C1[N+](=O)[O-]
Benzaldehyde	240	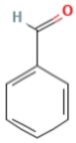	C1=CC=C(C=C1)C=O

Table S4. Compounds used in virtual screening in additional to those in Table S3.

Name	Pubchem CID	Name	Pubchem CID
Nonanoic acid	8158	Isopropyl Mercaptan	6364
Decanoic acid	2969	Undecanoic acid	8180
Heptanoic acid	8094	Pyrene	31423
Benzophenone	3102	Fluoranthene	9154
Ethyl isobutyrate	7342	Phenethyl acetate	7654
2-Methyl-1-propanethiol	10558	Undecanedioic acid	15816
Butyl formate	11614	β-ionone	638014
Alpha-terpinyl acetate	111037	Sebacid	5192
Butyl butyryl lactate	24114	1-Methylcyclopropene	151080
Methyl salicylate	4133	β-nadph tetrasodium salt	5884
Shoyu pyrazine	27458	1-Heptanethiol	15422
Isoamyl octanoate	16255	Oxoazelaic acid	269945
Dimethyl sulfide	1068	1-(methylthio)-octane	77289
Dextro-sorbitol	61431	Decane-1-10-dithiol	14494
2-Butanone	6569	2-methylbutane-1-thiol	15877
4-Methylvaleric acid	12587	Nonane-19-dithiol	248488
Ethylene brassylate	61014	Pentane-15-dithiol	70236
Guaiacol	460	3-Methyl-2-butanethiol	519823
Isovaleric acid	10430	1-Propanol	1031
Methanethiol	878	Isopropanol	3776
Pyrazine	9261	2-Methyl-2-propanol	6386
Terpineol	17100	2-butanol	6568
1-Hexanethiol	8106	(R)-(+)-1-Phenylethanol	637516
MTMT	122370	Phenetole	7674
NN-Dimethylethylamine	11723	Pyrrole	8027
2-Mercaptopyrimidine	1550489	Piperidine	8082
thioacetic acid	10484	β-Damascone	5374527
Trans-cyclo octene	5463599	Isopropyl tiglate	5367745
1-(methylthio)ethanethiol	525462	TNT	8376
(methylsulfanyl)methane	93236	26-bis(trimethylsilyl)benzenethiol	15376349
Cis-cyclooctene	638079	2-(Naphthyl)ethylamine hydrochloride	16218122
Hexanoic-66-D3 acid	12222599	N-methyl-2-phenylethylamine	11503
Methyl DL-lactate	11040	Sodium hydrosulfide	28015
Testosterone	6013	Curcumin	969516
2-Methyl-2-pentanethiol	74213	Trimethylamine	16387

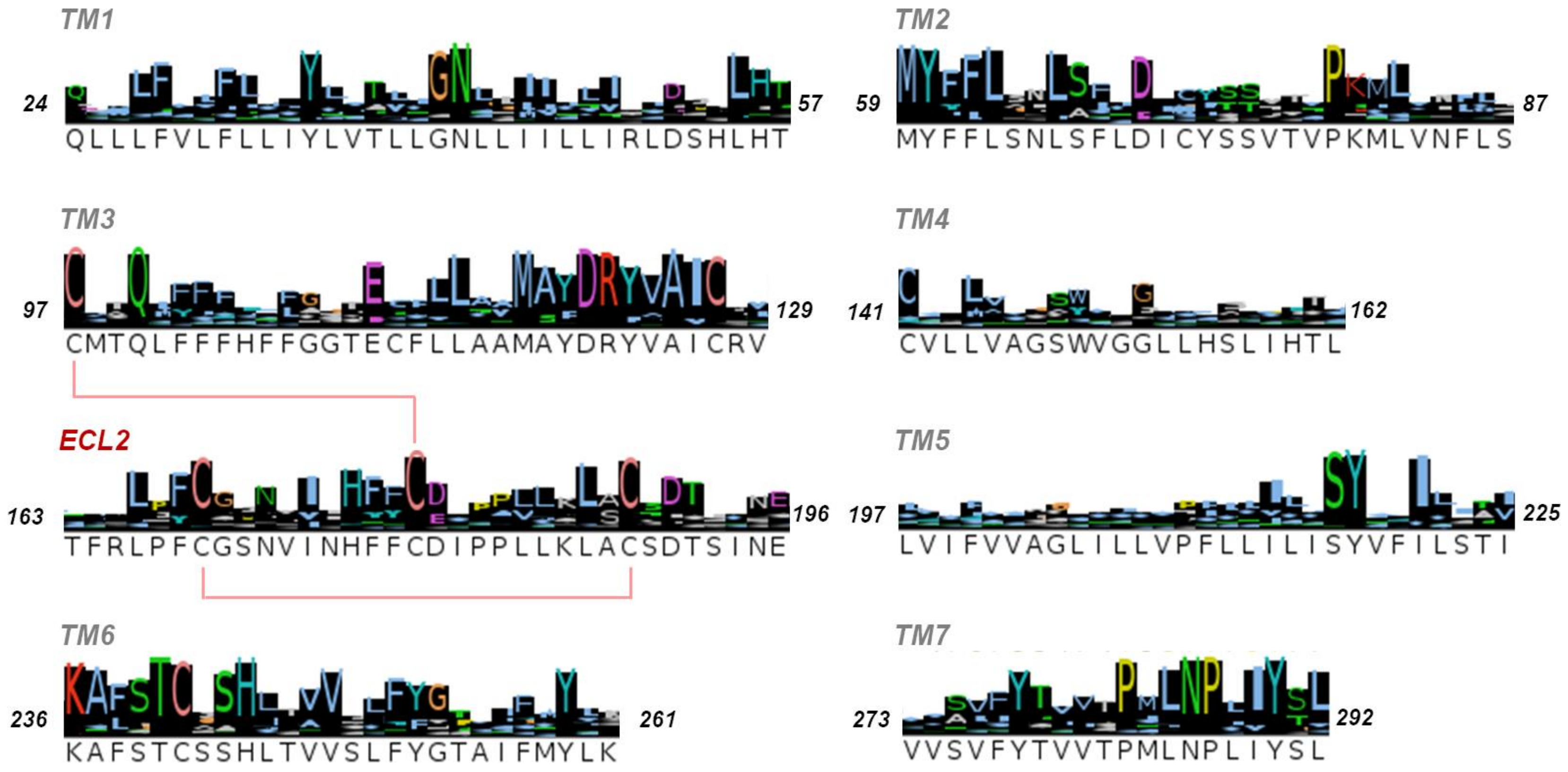

Figure S1. Consensus sequence of the TM regions and ECL2 in human and mouse ORs. Residue numbers in mOR256-3 are labeled on both sides of each region. Histogram indicates sequence conservation.

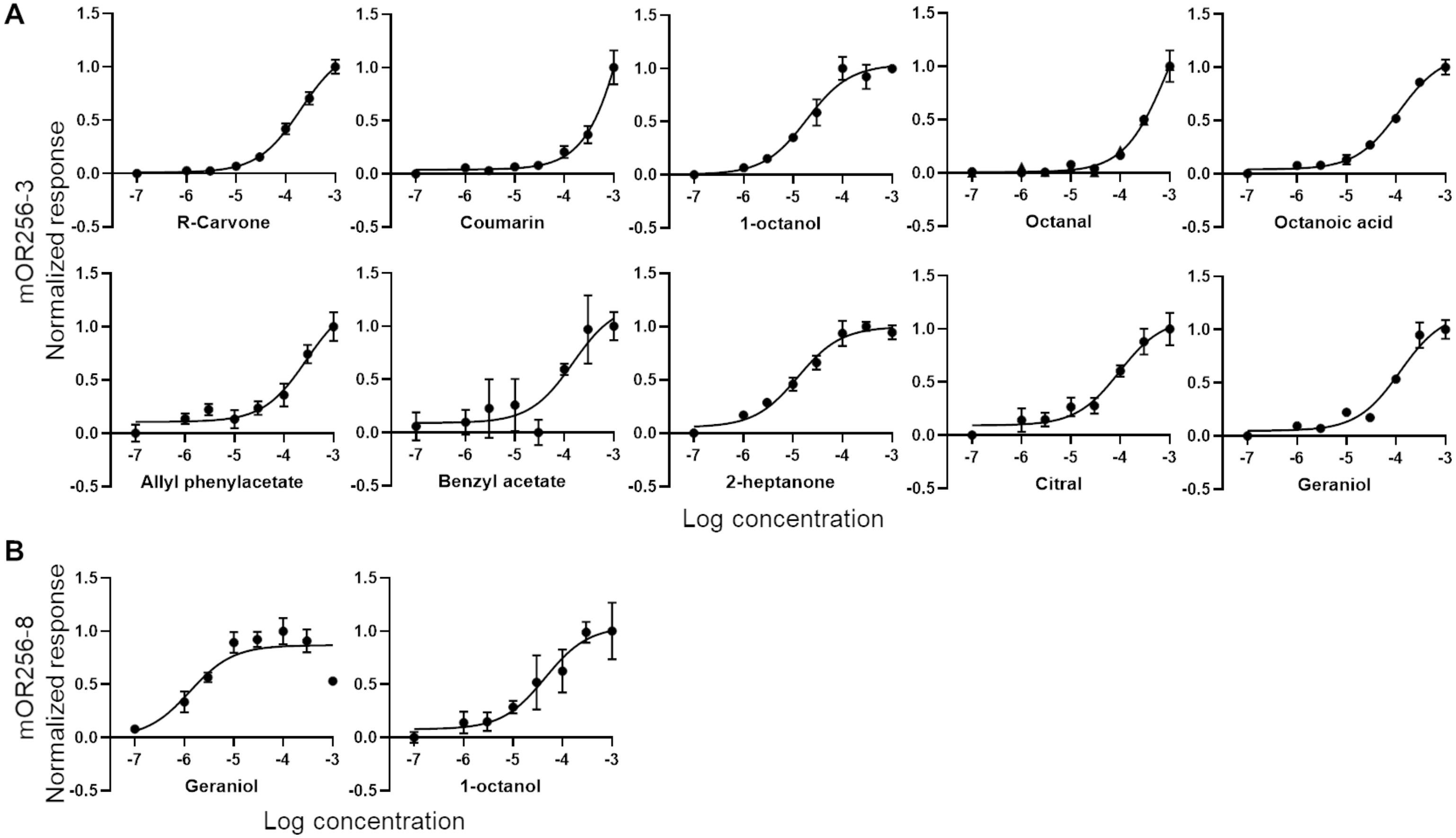

Figure S2. Dose-dependent response curves of **(A)** mOR256-3 and **(B)** mOR256-8 to their ligands. Data are mean ± SEM of 3 technical repeats.

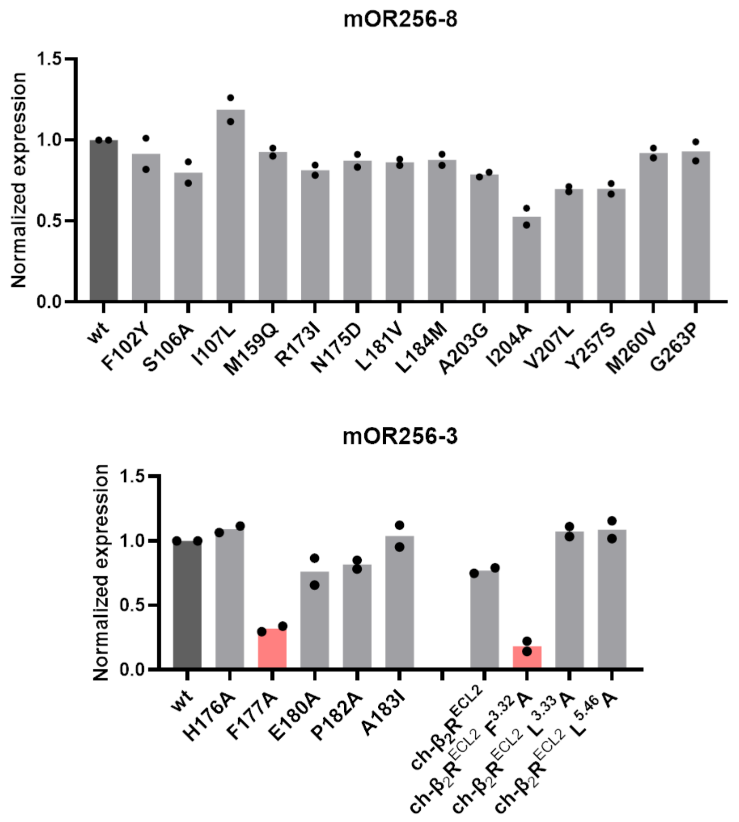

Figure S3. Cell-surface expression level of mOR256-3 and mOR256-8 variants relative to the wt receptor. Two technical repeats were performed.

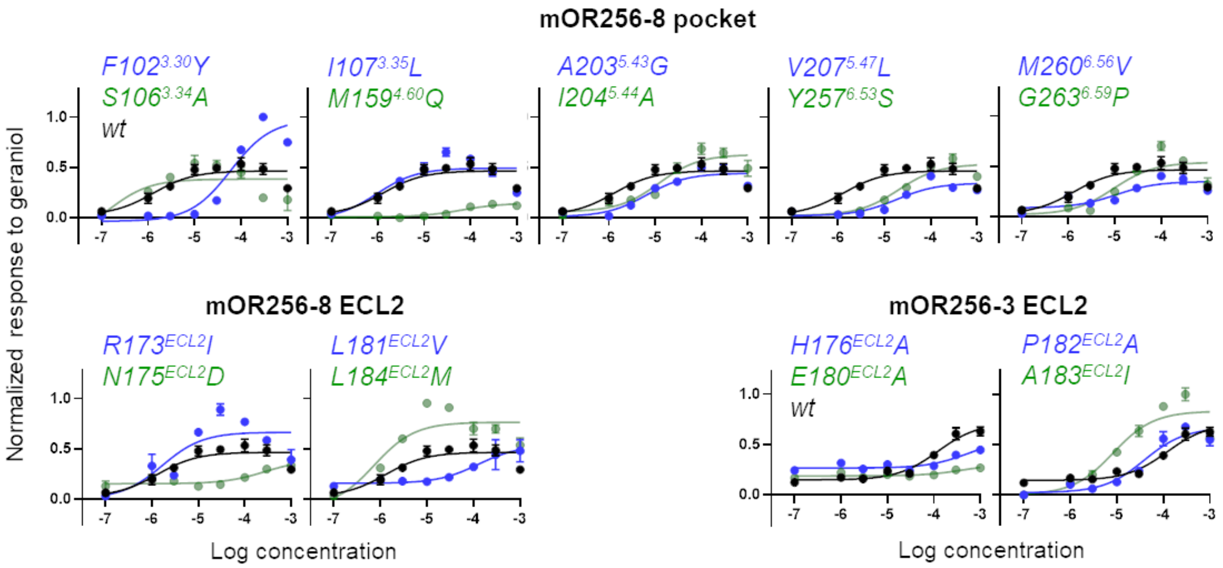

Figure S4. Dose-dependent response curves of mOR256-3 and mOR256-8 variants to geraniol. Data are mean ± SEM of 3 technical repeats.

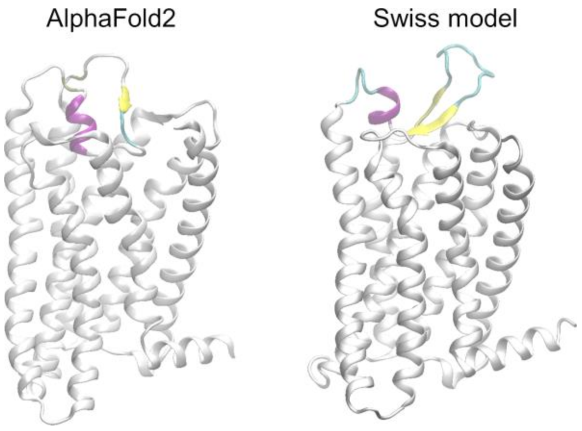

Figure S5. mOR256-3 models built by AlphaFold2 and Swiss model. ECL2 is colored by secondary structures. The N- and C-termini are neglected.

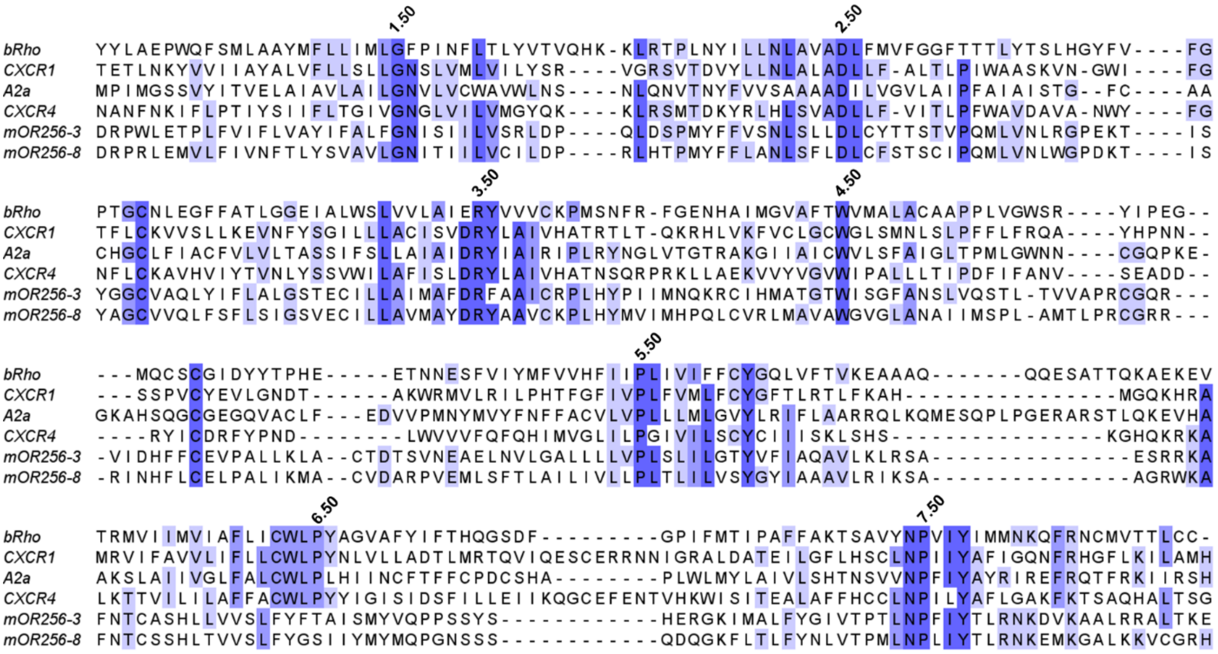

Figure S6. Sequence alignment for the homology modeling of mOR356-3 and mOR256-8.

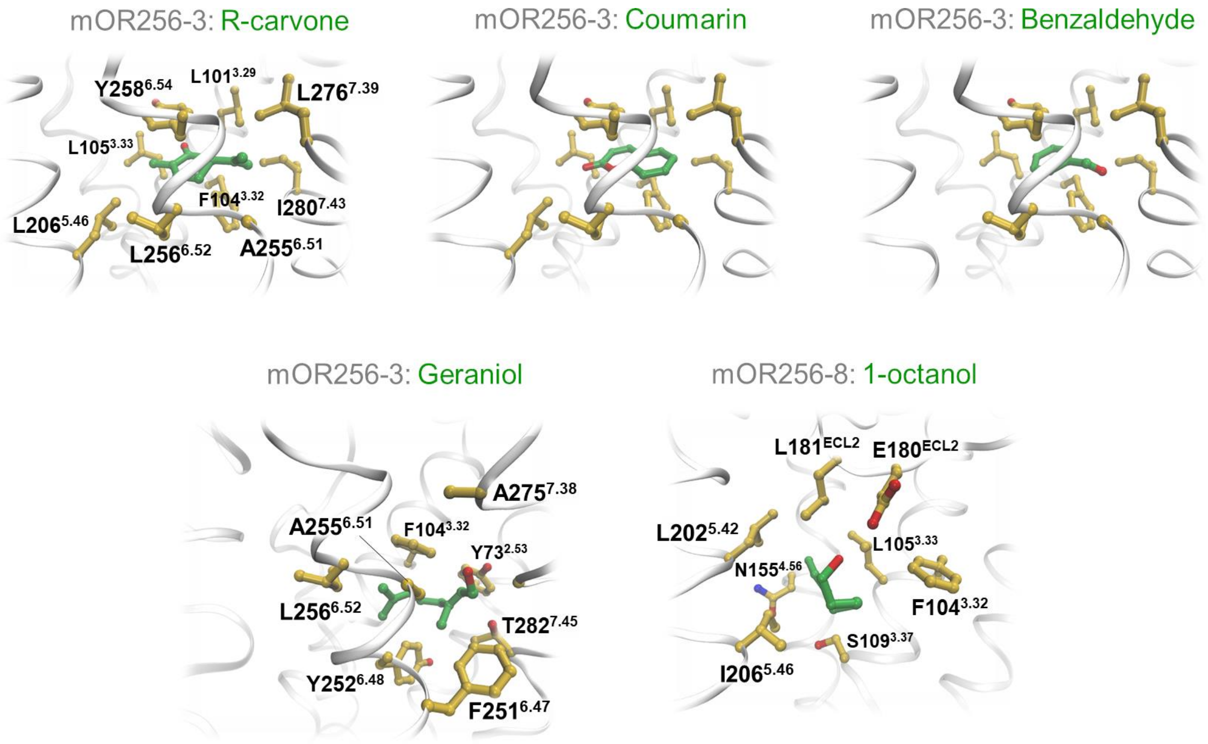

Figure S7. Predicted ligand interactions with mOR256-3 and mOR256-8.

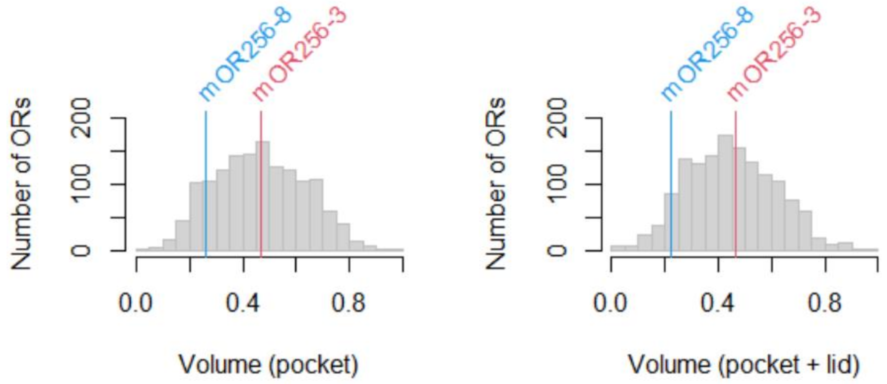

Figure S8. Histogram of normalized pocket volume of human and mouse ORs. The pocket volume was calculated from the sum of the side-chain volume of the residues forming the pocket and the lid. We used the 17 pocket residues identified in our previous work (34), in addition to the lid residues chosen according to the homology model in this work. The same residues were used for all the ORs according to the sequence alignment. It is a coarse estimation assuming similar shape and side-chain orientations in the pocket, without considering the 3D stacking of the residues.

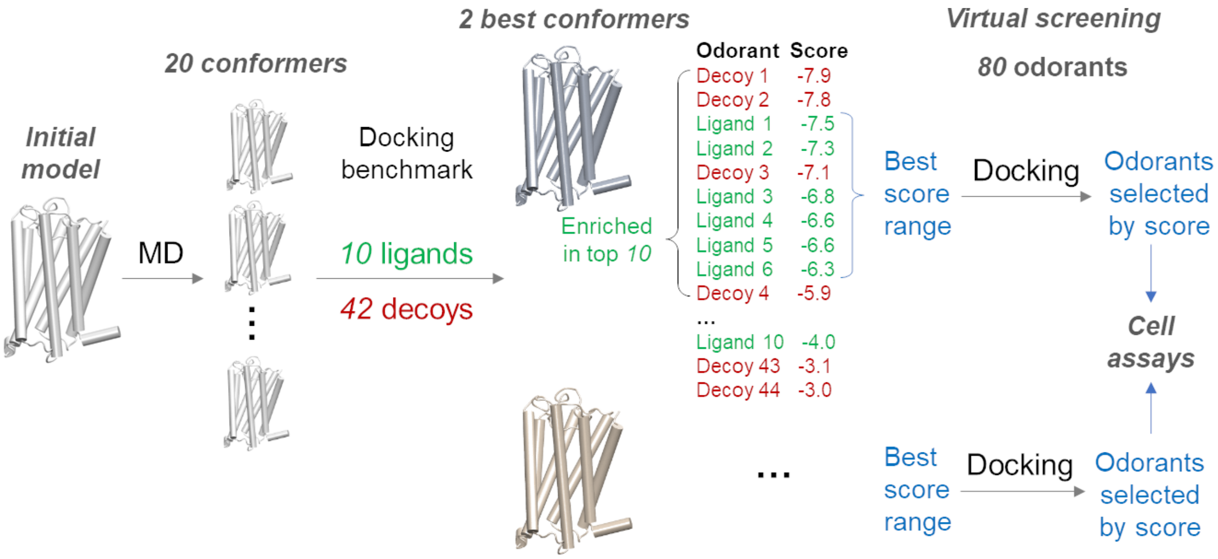

Figure S9. Virtual screening protocol. MD simulations were first performed on the initial model to obtain 20 conformers. Benchmark compounds were docked to each of the conformers and ranked by docking scores for the given conformer. The best conformers were chosen as those that returned the most ligands in the 10 top-ranked compounds. The range of scores that best separated the ligands from the decoys was used in the subsequent virtual screening to select hits. We used 2 best conformers for virtual screening and the common hits were tested in cell assays.

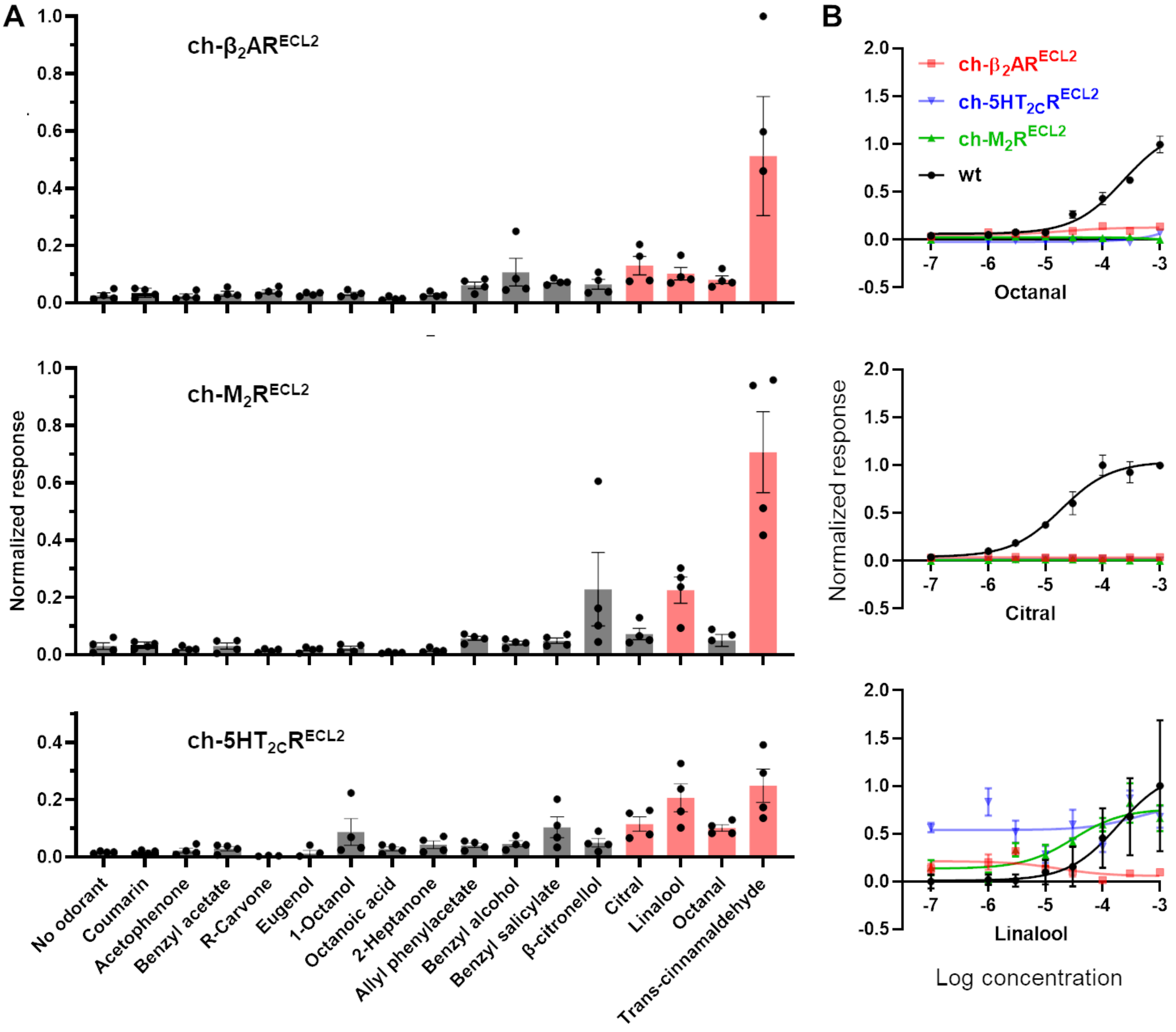

Figure S10. Functional assays of mOR256-3 chimeras. (**A**) Screening of 16 odorants at 300 μM concentration. Significant responses are colored in red, which were tested in dose-dependent assays in (**B**). Data are mean ± SEM of 3-4 technical repeats.
